# *Ab initio* random structure searching of organic molecular solids: assessment and validation against experimental data†
†Electronic supplementary information (ESI) available: Results of similarity analysis between the 11 structures of lowest energy obtained in the AIRSS calculations and the reported structures of form III and form IV of *m*-ABA; unit cell parameters and volumes for all structures considered; comparison of 2*θ* values derived from the unit cell parameters of different structural models representing form III of *m*-ABA; Le Bail fitting of the experimental powder XRD pattern of form IV of *m*-ABA recorded at 70 K using, as the initial structural model, the reported crystal structure following geometry optimization; table of calculated (GIPAW) absolute isotropic NMR shieldings; simulated powder XRD data for the considered structures after precise geometry optimization; experimental ^1^H MAS NMR spectra of forms III and IV. (pdf) The calculated and experimental data for this study are provided as a supporting dataset from WRAP, the Warwick Research Archive Portal at http://wrap.warwick.ac.uk/91884. See DOI: 10.1039/c7cp04186a


**DOI:** 10.1039/c7cp04186a

**Published:** 2017-09-25

**Authors:** Miri Zilka, Dmytro V. Dudenko, Colan E. Hughes, P. Andrew Williams, Simone Sturniolo, W. Trent Franks, Chris J. Pickard, Jonathan R. Yates, Kenneth D. M. Harris, Steven P. Brown

**Affiliations:** a Department of Physics , University of Warwick , Coventry CV4 7AL , UK . Email: S.P.Brown@warwick.ac.uk; b School of Chemistry , Cardiff University , Park Place , Cardiff CF10 3AT , UK . Email: HarrisKDM@cardiff.ac.uk; c Scientific Computing Department , Rutherford Appleton Laboratory , Chilton , Didcot , Oxfordshire OX11 0QX , UK; d Department of Materials Science & Metallurgy , University of Cambridge , 27 Charles Babbage Road , Cambridge CB3 0FS , UK; e Department of Materials , University of Oxford , Oxford OX1 3PH , UK . Email: Jonathan.Yates@materials.ox.ac.uk

## Abstract

This paper explores the capability of using the DFT-D *ab initio* random structure searching (AIRSS) method to generate crystal structures of organic molecular materials, focusing on a system (*m*-aminobenzoic acid; *m*-ABA) that is known from experimental studies to exhibit abundant polymorphism. Within the structural constraints selected for the AIRSS calculations (specifically, centrosymmetric structures with *Z* = 4 for zwitterionic *m*-ABA molecules), the method is shown to successfully generate the two known polymorphs of *m*-ABA (form III and form IV) that have these structural features. We highlight various issues that are encountered in comparing crystal structures generated by AIRSS to experimental powder X-ray diffraction (XRD) data and solid-state magic-angle spinning (MAS) NMR data, demonstrating successful fitting for some of the lowest energy structures from the AIRSS calculations against experimental low-temperature powder XRD data for known polymorphs of *m*-ABA, and showing that comparison of computed and experimental solid-state NMR parameters allows different hydrogen-bonding motifs to be discriminated.

## Abstract 2

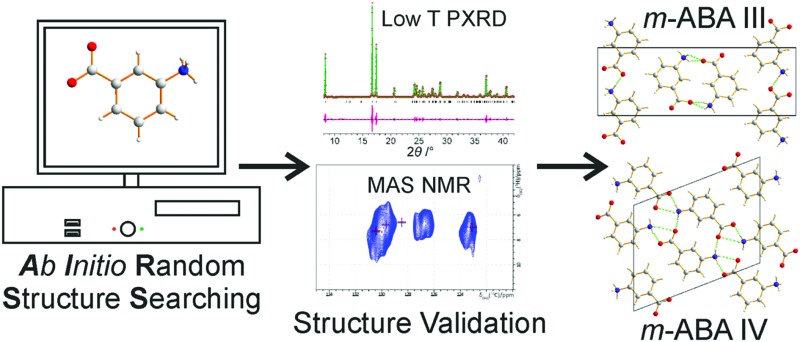
The AIRSS method generates crystal structures for *m*-aminobenzoic acid; comparison is made to experimental powder X-ray diffraction and MAS NMR.

## Introduction

Determination of the three-dimensional arrangement of molecules in crystalline organic materials – the “crystal structure” – is an essential pre-requisite for understanding and rationalizing the physicochemical properties of these materials. If single crystals of sufficient size and quality can be prepared for the material of interest, determination of the crystal structure by single-crystal X-ray diffraction (XRD) is nowadays very routine. Even if single crystals of sufficient size and quality for single-crystal XRD are not available, structure determination of organic materials directly from powder XRD data is now (since the early 1990s) a viable technique.^1^–^11^ However, cases still arise for which structure determination proves to be elusive. For example, the crystal structure of one of the known polymorphs of *m*-aminobenzoic acid (*m*-ABA) has not yet been determined,^12^ as discussed in more detail below.

In addition to experimental methods for characterizing polymorphs^13^–^21^ of organic molecules, such as powder XRD and solid-state NMR, a variety of computational approaches allow crystal structure landscapes to be explored for a molecule of interest, leading to knowledge of the polymorphs that could, in principle, be experimentally accessible. Such crystal structure prediction (CSP) approaches are being developed and applied by several groups, leading to increased success in the reliability of predicting crystal structures of organic molecules.^19^,^22^–^31^

In the present work, we explore the application of the *ab initio* random structure search (AIRSS) method^32^,^33^ for crystal structure prediction of organic materials. As input parameters, AIRSS only requires the specification of the atoms and/or molecular unit present in the structure, the number of formula units in the unit cell (*Z*) and the approximate density. Other chemically and structurally intuitive constraints can also be applied, such as minimum interatomic distances between particular types of atom and specific symmetry operations. We emphasize that no energy calculations are actually involved in generating the initial trial structures in AIRSS. Subsequently, within the AIRSS approach used here, each trial structure is subjected to geometry optimization by carrying out a full periodic DFT calculation, leading to an energy ranking of all structures generated.

The AIRSS method has been applied to several different classes of material, focused primarily on inorganic materials, yielding new insights into structure formation under high pressure,^34^,^35^ battery materials^36^–^38^ and minerals.^39^ In the case of organic molecular materials, the application of AIRSS (with fixed unit-cell dimensions) has been demonstrated^33^ for the dipeptide β-AspAla and calculations using AIRSS were included in the 5th and 6th blind tests of crystal structure prediction organized by the Cambridge Crystallographic Data Centre.^40^,^41^ We note that the first principles approach used here, in which trial structures generated within AIRSS are subjected to energy minimization using periodic DFT calculations, is not currently competitive with the bespoke CSP approaches commonly applied for organic molecular crystals.^19^,^22^–^31^ The feasibility of exploring vast numbers of trial crystal structures will improve with the ever increasing power of computational resources in the long term. The AIRSS approach only requires minimal parameters and does not need force fields to be constructed, and thus it is likely to find unusual, but physically feasible, structural features that may be missed by other methods. The complementarity to existing methods suggests that AIRSS should become a widely adopted approach.

Crystal structure prediction methods, such as AIRSS, produce a large number of trial structures, generally with the aim of discovering new (experimentally unknown) polymorphs of the molecule of interest, which may have specific desirable properties. Within this endeavour, it is also important to establish whether any of the predicted structures correspond to polymorphs that are already known experimentally, for example by comparing simulated powder XRD patterns for the predicted structures with powder XRD data for the experimentally known polymorphs. Although, in principle, this task is straightforward, it may be challenging in certain cases, particularly as experimental powder XRD data are usually recorded at ambient temperature, whereas energy minimization calculations carried out as part of the structure prediction process effectively deliver structural information at *T* = 0 K. The simulated and experimental powder XRD patterns may differ significantly in appearance, particularly in regions with significant peak overlap, as a consequence of these differences in lattice parameters due to temperature differences between a predicted structure (effectively at *T* = 0 K) and the experimental powder XRD data (typically ambient temperature).

In principle, solid-state NMR provides another opportunity to assess whether predicted crystal structures match a particular experimental sample. In the NMR crystallography approach,^42^–^47^ NMR parameters can be calculated for a predicted crystal structure using DFT and the GIPAW method. The calculated NMR parameters can then be compared with experimental solid-state NMR data. The main barrier to uniquely identifying a structure using NMR alone is the “error” (*i.e.*, discrepancy as compared to experiment) in calculating NMR parameters by DFT methods, which, in some cases, can be comparable to the difference in NMR parameters between polymorphs (particularly for polymorphs in which the molecules have similar local environments).

**Scheme 1:**
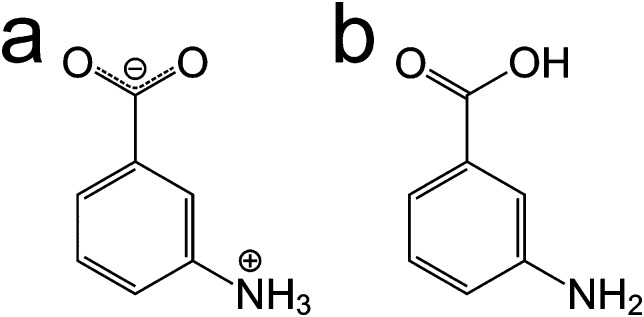
The *m*-ABA molecule in (a) the zwitterionic form and (b) the non-zwitterionic form.

In spite of the specific challenges associated with each of these approaches, a careful combination of computational modelling, powder XRD and solid-state NMR can enhance the prospects for determining the crystal structures of materials. In this paper, we explore several aspects relating to the combined use of these techniques, focusing on *m*-ABA, for which there are currently five known polymorphs.^12^ In three polymorphs (forms I, III and IV), the molecule is zwitterionic (Scheme 1a). In the other two polymorphs (forms II and V), the molecule is non-zwitterionic (Scheme 1b). The crystal structure of form II (CSD Refcode: AMBNZA) has been determined^48^ by single-crystal XRD, and the crystal structures of form III (CSD Refcode: AMBNZA01), form IV (CSD Refcode: AMBNZA02) and form V (CSD Refcode: AMBNZA03) have been determined^12^ directly from powder XRD data. The crystal structure of form I has not yet been determined, due to challenges in indexing the powder XRD data, which has unusual peak shapes and excessive peak overlap. However, it is known from X-ray photoelectron spectroscopy studies^12^ and high-resolution solid-state ^13^C NMR studies^49^ that form I contains zwitterionic *m*-ABA molecules. In forms II and V, the non-zwitterionic molecules form carboxylic acid “dimers” (linked by two O–H···O hydrogen bonds), whereas in forms III and IV, the zwitterionic molecules are linked by N–H···O hydrogen bonds between the NH_3_^+^ and COO^–^ groups of neighbouring molecules.

This paper presents the results of an AIRSS search for energetically accessible crystal structures containing *m*-ABA molecules in the zwitterionic form in centrosymmetric crystal structures with *Z* = 4. In part, these conditions were selected in order to assess the ability of AIRSS to find the crystal structures of form III and form IV, as well as (potentially) to find other energetically accessible polymorphs that have not yet been observed experimentally. Importantly, our work explores issues concerning the assessment and validation of results from crystal structure prediction by considering experimental powder XRD data and solid-state NMR data, including ^1^H–^13^C two-dimensional magic-angle spinning (MAS) NMR and GIPAW^45^,^50^–^52^ calculation of NMR chemical shifts.

## Computational and experimental details

### Crystal structure prediction

The AIRSS approach used here involves two stages: first, trial structures are generated by the random search component and, second, each of these trial structures is then subjected to geometry optimization using a full periodic DFT calculation (see next section), leading to an energy-ranked list of structures. In the present work to generate crystal structures of *m*-ABA using the AIRSS method,^32^,^33^ the first (random search) stage of the calculation specifically used *m*-ABA molecules in the zwitterionic form. The number of molecules in the unit cell was constrained to *Z* = 4 and space group *P*1 was imposed. These conditions were selected as the two polymorphs containing zwitterionic *m*-ABA molecules and with known crystal structures (forms III and IV) have *Z* = 4 and are centrosymmetric. Thus, our calculations allow an assessment of the ability of AIRSS to successfully find these two known polymorphs.

In the first stage of the calculation, the unit cell parameters were allowed to vary subject to a target volume per molecule and the unit cell angles were restricted to the range 70° to 110°. A feature of this stage of AIRSS calculations is that minimum intermolecular atom–atom distances may be specified in order to eliminate unreasonable structures (*e.g.*, in which atoms approach too close to each other) or to bias the search towards (or away from) structures containing specific types of intermolecular interaction. Table 1 lists the shortest intermolecular atom–atom distances in the known crystal structures of the zwitterionic polymorphs (forms III and IV) of *m*-ABA, together with the minimum distances allowed in the first stage of our AIRSS search. With the exception of the minimum N···O and O···O distances, the minimum distances used in the AIRSS search were chosen, somewhat arbitrarily, to be between 80 and 90% of the shortest intermolecular atom–atom distance of the relevant type in the known crystal structures of the zwitterionic polymorphs (forms III and IV) of *m*-ABA. Future research will consider the optimum choices for the different minimum distances used in AIRSS searches for organic molecular crystals. We note that restricting the shortest allowed intermolecular O···O distance to 3.0 Å ensures that no short intermolecular O···O distances are formed, which are implausible on electrostatic grounds for zwitterionic *m*-ABA molecules.

**Table 1:** Shortest intermolecular atom–atom distances (Å) in the reported crystal structures of zwitterionic polymorphs of *m*-ABA and the distance constraints imposed in the first stage (random search) of the AIRSS calculations

	H···H	H···C	H···N	H···O	C···C	C···N	C···O	N···N	N···O	O···O
Form III	2.38	2.72	3.10	1.89	3.59	3.24	3.25	3.57	2.78	3.21
Form IV	2.17	2.54	3.08	1.77	3.52	3.19	3.18	3.52	2.67	3.32
Distance constraints in AIRSS	1.7	2.0	2.7	1.6	2.8	1.7	2.6	3.2	2.6	3.0

We emphasize that the first stage of the AIRSS search was carried out using the *m*-ABA molecule in the zwitterionic form, therefore guaranteeing that all initial trial crystal structures are zwitterionic. The use of the minimum distance constraints discussed above serves to discourage the AIRSS search from generating trial structures that would correspond to implausible intermolecular contacts for zwitterionic molecules.

### First-principles calculations

DFT calculations were carried out using the CASTEP code,^53^ which uses a planewave basis-set together with pseudopotentials to represent the core–valence interaction. All calculations used the PBE functional^54^ together with the DFT-D dispersion correction scheme of Tkatchenko-Scheffler.^55^ The initial DFT calculation carried out on the trial structures generated in the first stage of the AIRSS search used CASTEP geometry optimization with a planewave cut-off energy of 500 eV and a Brillouin zone sampling of 2π × 0.1 Å^–1^. The convergence criteria were 0.01 eV Å^–1^ for forces, 0.01 GPa for stresses, 0.00001 eV per atom for energy and 0.001 Å for atomic displacements. Performance was improved by running the calculations under external pressure (with a stress of 0.5 GPa applied to the structure), which can be considered to constrain the separation of the molecules. The DFT calculations carried out in the second stage of the AIRSS search under these conditions are described as initial geometry optimization.

The structures of lowest enthalpy were then subjected to further optimization using a stricter set of parameters, which we define as precise geometry optimization. The parameters for these calculations were: zero external pressure, a planewave cut-off energy of 800 eV, Brillouin zone sampling of 2π × 0.05 Å^–1^, and convergence criteria of 0.000005 eV per atom for changes in energy and 0.0005 Å for atomic displacements. These precise calculations used the CASTEP 8.0 set of on-the-fly pseudopotentials.^56^ To allow comparison of unit cell parameters, all structures generated in AIRSS were converted to conventional unit cell representations.^57^

Magnetic shieldings were calculated using the GIPAW approach^45^,^50^–^52^ with the CASTEP 8.0 set of on-the-fly pseudopotentials, a plane-wave cut-off energy of 800 eV and Brillouin zone sampling of 2π × 0.05 Å^–1^. To analyse the large amount of computed NMR parameters, MagresPython libraries were used.^58^

### Sample preparation

Polycrystalline samples of form III and form IV of *m*-ABA were prepared using the procedures described previously.^12^ Form III was used as purchased from Aldrich and form IV was obtained by sublimation and condensation onto a glass cold finger at ambient temperature.

### Experimental powder XRD

Ambient-temperature powder XRD data were recorded using a Bruker D8 instrument (Cu Kα_1_, Ge monochromated) in transmission geometry, with data collected in the range 4° ≤ 2*θ* ≤ 70° (step size, 0.017°). For form III of *m*-ABA, the data were recorded with the sample contained between two pieces of tape in a foil-type sample holder (time per step, 12 s; total data collection time, 13 h 29 m). For form IV, the data were recorded with the sample in a capillary (time per step, 15 s; total data collection time, 16 h 52 m). Low-temperature powder XRD data were recorded for form III and for form IV at 70 K on a Bruker D8 instrument (Cu Kα_1_, Ge monochromated) in reflection geometry using an Oxford Cryosystem Phenix temperature controller. The data were recorded in the range 4° ≤ 2*θ* ≤ 43° (step size, 0.016°; time per step, 8 s; total data collection time, 5 h 50 m). For each sample, three powder XRD patterns were recorded consecutively using the parameters described above, and were then summed. Le Bail fitting^59^ of the powder XRD data was carried out using the GSAS program.^60^

### Experimental solid-state NMR

Solid-state NMR experiments were carried out at ambient temperature using a 14.1 *T* (^1^H Larmor frequency, 600 MHz) Bruker Avance II+ spectrometer equipped with a Bruker 1.3 mm HXY probe (operating in double resonance mode). For rf pulses (not during cross polarization (CP) or decoupling), the 90° (^1^H) pulse duration was 2.5 μs. ^1^H and ^13^C chemical shifts are referenced indirectly to tetramethylsilane (TMS) using the methyl signals of l-alanine at 1.3 ppm (^1^H) and 20.5 ppm (^13^C), which correspond to 1.85 ppm (^1^H) and 38.5 ppm (^13^C) for adamantane.^61^ For the 2D ^1^H–^13^C experiments, a tangential ramp^62^ at ^1^H and ^13^C nutation frequencies of 100 and 40 kHz, respectively, was applied at a resonance offset corresponding to 5.4 and 103 ppm in the ^1^H and ^13^C dimensions, respectively. ^1^H decoupling was applied during acquisition of the ^13^C FID, using swept low-power two-pulse phase modulation (slp TPPM)^63^ at a nutation frequency of 20 kHz. A nested 8-step phase cycle was used to select a change in coherence order (*p*) of Δ*p* = ±1 on the 90° (^1^H) pulse, with the phase of the ^13^C CP pulse cycled through [+*x*, –*x*, +*y*, –*y*] and with the receiver phase following.

## Results

**Fig. 1:**
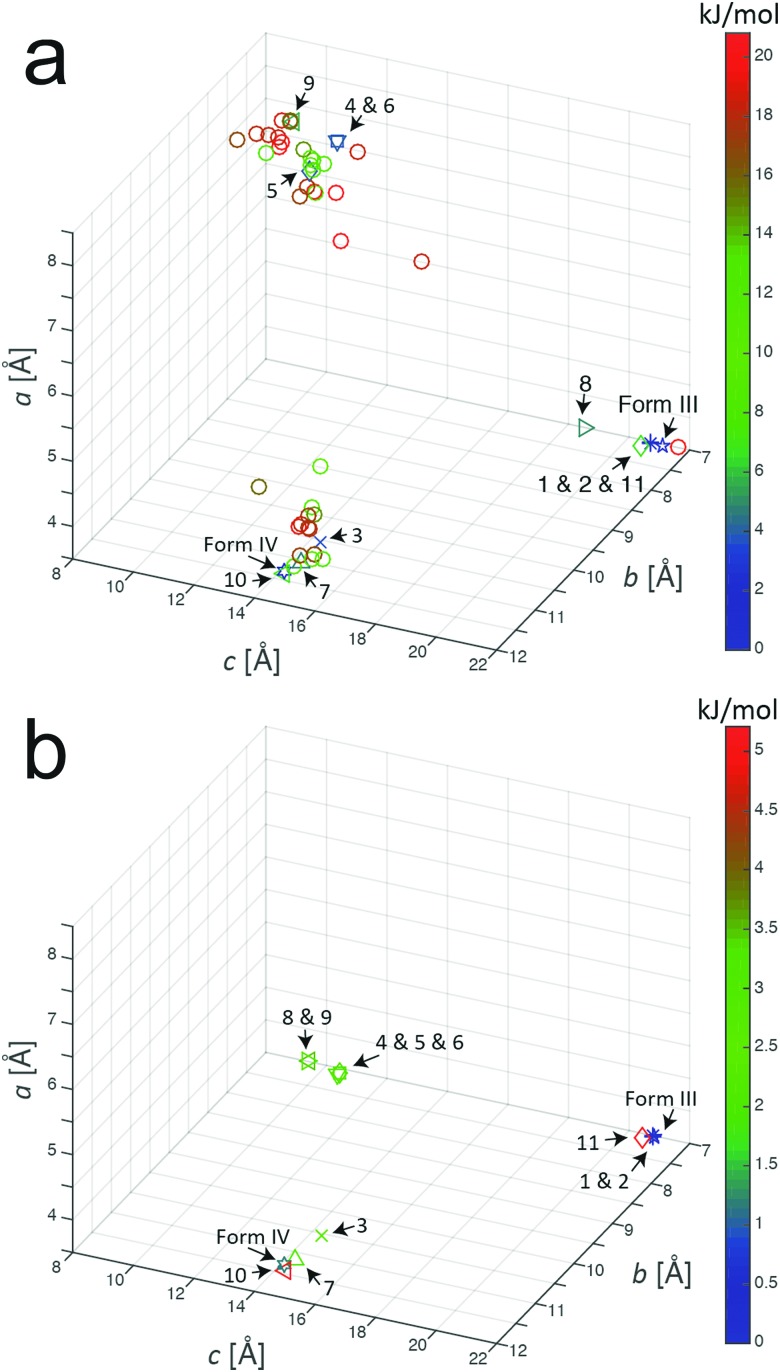
Unit cell parameters for structures resulting from the AIRSS calculation on *m*-ABA (after converting to conventional unit cell settings) for: (a) the 50 lowest-energy structures generated from AIRSS using initial geometry optimization, and (b) the 11 lowest-energy structures after subsequent precise geometry optimization (the colour of the label for each structure indicates the energy relative to the structure of lowest energy). The unit cell parameters for the reported structures of form III and form IV of *m*-ABA (after the precise geometry optimization procedure) are also shown. All unit cell parameters are listed in Table S2 (ESI†).

Our AIRSS search on *m*-ABA generated 600 structures and the unit cells of these structures following the initial geometry optimization stage of the AIRSS calculation were transformed into conventional unit cells.^57^ Initially, we focused on the 50 structures of lowest energy (which spanned an energy range of 20.8 kJ mol^–1^). Fig. 1a shows the unit cell lengths *a*, *b* and *c* for these 50 structures, together with the unit cell lengths for the reported structures of forms III and IV after subjecting these structures to the same geometry optimization procedure (including relaxation of unit cell parameters). From Fig. 1a, it appears that the 50 structures of lowest energy from AIRSS fall into three broad clusters on the basis of unit cell dimensions. Within one cluster (including predicted structures **1**, **2**, **8** and **11**, numbered according to their ranking by energy), the unit cell dimensions are similar to the known crystal structure of form III. For another cluster (including predicted structures **3**, **7** and **10**), the unit cell dimensions are similar to the known crystal structure of form IV.

A recent analysis^64^ of over 1000 experimentally determined crystal structures, including over 500 polymorphs of organic molecules, concluded that, in 95% of cases, the difference in energy between experimentally observed polymorphs is less than 7.2 kJ mol^–1^. On this basis, the present paper assumes that any structure found in the AIRSS search for which the calculated energy (following initial geometry optimization) is within 7.2 kJ mol^–1^ of the structure of lowest energy found in the search is considered to be an “experimentally accessible” polymorph (note: 1 kJ mol^–1^ = 0.0104 eV per molecule). From our AIRSS calculation on *m*-ABA, 11 structures were obtained within this 7.2 kJ mol^–1^ range. A further precise geometry optimization was then carried out for each of these 11 structures, and the resulting unit cell lengths *a*, *b* and *c* are shown in Fig. 1b. For each of these structures, the energy (both for initial and precise geometry optimizations), the unit cell volume (per molecule) and space group are given in Table 2. The corresponding information for the reported crystal structures of forms III and IV (following the same initial and precise geometry optimization procedures) are also shown in Table 2.

**Table 2:** Data for the 11 lowest-energy crystal structures of *m*-ABA generated by AIRSS: energy (relative to the structure of lowest energy (structure **1**)), volume per molecule and space group

Structure	Space group	*Z*′	*Z*	Structure after initial geometry optimization		Structure after precise geometry optimization	
				Energy (kJ mol^–1^)	Volume per molecule (Å^3^)	Energy (kJ mol^–1^)	Volume per molecule (Å^3^)
**1**	*P*2_1_/*c*	1	4	0.00	142.61	0.00	144.43
Form III^*a*^	*P*2_1_/*c*	1	4	0.10	142.52	0.00	144.43
**2**	*P*2_1_/*c*	1	4	0.68	142.49	0.00	144.40
Form IV^*a*^	*P*1	2	4	1.64	144.13	1.16	145.96
**3**	*P*1	2	4	3.57	146.04	1.06	148.32
**4**	*P*1	1	2^*c*^	3.76	144.81	2.12	147.17
**5**	*P*1	1	2^*c*^	4.15	144.97	2.22	146.99
**6**	*P*1	1	2^*c*^	4.15	144.65	2.12	146.89
**7**	*P*1	2	4	4.82	146.26	2.03	148.26
**7_R_** ^*b*^	*P*1	2	4	—	—	1.16	146.10
**8**	*P*1	1	2^*c*^	5.11	150.86	1.93	154.01
**9**	*P*1	1	2^*c*^	5.69	150.76	2.22	153.82
**10**	*P*1	2	4	6.75	147.74	3.96	149.95
**11**	*P*1	2	4	6.85	147.98	5.21	150.37

^*a*^Results for forms III and IV of *m*-ABA were obtained by subjecting the reported crystal structures to the same geometry optimization procedures (with relaxation of unit cell parameters) used for the structures generated by AIRSS. The experimental unit cell volumes^12^ (at ambient temperature) are 146.50(1) Å^3^ per molecule for form III and 148.78(1) Å^3^ per molecule for form IV. The published structure of form III (space group *P*2_1_/*a*) has been transformed here to the conventional unit cell (for which the space group is *P*2_1_/*c*).

^*b*^Structure **7_R_** was generated from structure **7** by rotating the H atoms of the C–NH_3_^+^ group by 60° around the C–N bond prior to geometry optimization (see discussion in the text).

^*c*^For structures **4**, **5**, **6**, **8** and **9**, the AIRSS search with *Z* = 4 represented a superstructure (with the unit cell volume doubled by translation) of a structure with *Z* = 2 and space group *P*1. In these cases, the crystal structure is described using the true (*Z* = 2) unit cell (note that this description is adopted in Fig. 1b).

While *P*1 symmetry was imposed in generating trial structures in AIRSS, subsequent inspection revealed that the actual space group of predicted structures **1** and **2** (following geometry optimization) was *P*2_1_/*c*. In the case of structures **4**, **5**, **6**, **8** and **9**, the structure with *Z* = 4 generated by AIRSS actually represented a superstructure (with the unit cell volume doubled by translation) of a structure with *Z* = 2 and space group *P*1. In these cases, the crystal structure is described subsequently using the true (*Z* = 2) unit cell; this description is used in Fig. 1b, from which it is clear that the length of the *a*-axis of the unit cell is very similar (between 3.7 and 4.1 Å, see Table S2, ESI†) in all 11 structures generated by AIRSS. We note that an alternative approach to assess the similarity between structures generated by AIRSS and the reported crystal structures of forms III and IV is to use the COMPACK crystal structure similarity procedure^65^ (analysis using this method is shown in Table S1 of the ESI†).

Before examining the lowest-energy structures from AIRSS, we consider in general terms the most appropriate method for comparing powder XRD data simulated for predicted structures (generated by AIRSS and following precise geometry optimization) and powder XRD data recorded experimentally for known solid forms. We begin by considering the reported structure^12^ of form III of *m*-ABA, for which the unit cell volume determined at ambient temperature is 146.50 Å^3^. Subjecting this crystal structure to the initial geometry optimization procedure (at 0.5 GPa), the resulting unit cell volume is 142.52 Å^3^. Then, following the subsequent precise geometry optimization procedure, the unit cell volume increases to 144.43 Å^3^. The fact that the unit cell volumes obtained following geometry optimization are lower than the unit cell volume of the experimentally determined structure is a consequence of the neglect of thermal effects in the geometry optimization calculations. In powder XRD data, the peak positions (2*θ* values) depend on the unit cell parameters, and therefore the set of peak positions in powder XRD data calculated for a predicted structure (nominally representing the structure at *T* = 0 K) are intrinsically different from the peak positions in experimental powder XRD data recorded for the same polymorph at temperatures above *T* = 0 K.^66^

**Fig. 2:**
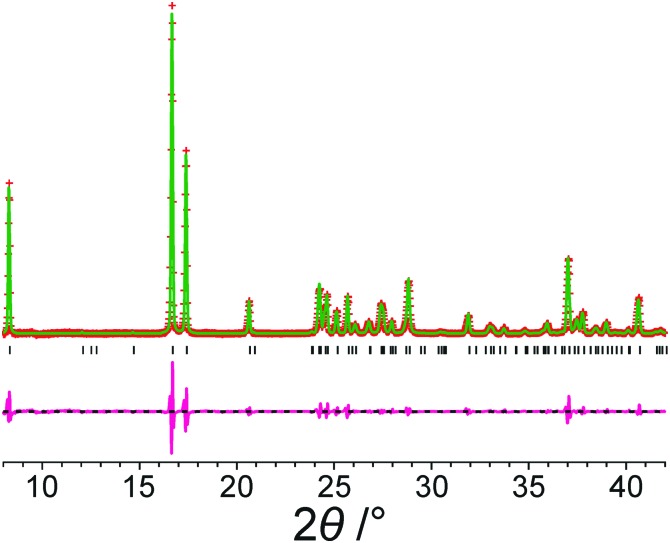
Le Bail fit (red + marks, experimental data; green line, calculated data; magenta line, difference plot; black tick marks, predicted peak positions) of an experimental powder XRD pattern (recorded at 70 K) of form III of *m*-ABA starting from the structure obtained following precise geometry optimization of the reported crystal structure of form III (see Table 2). The unit cell parameters obtained are listed in Table S2, ESI†.

Fig. 2 shows the results from Le Bail fitting of experimental powder XRD data recorded for form III of *m*-ABA at 70 K using, as input to the fitting procedure, the unit cell of the structure obtained following precise geometry optimization of the reported crystal structure of form III. Clearly, a good quality Le Bail fitting is achieved (the calculated 2*θ* values from this Le Bail fitting procedure are listed in Table S3, ESI†). In contrast, Le Bail fitting of experimental powder XRD data recorded at ambient temperature and using the same unit cell (from the geometry optimization) as input was not successful. As shown in Table 3, the unit cell of the structure of form III resulting from precise geometry optimization is very similar to the unit cell obtained from the Le Bail fitting of the powder XRD data recorded at low temperature (70 K), with a difference in unit cell volume of 0.3%, but differs significantly from the unit cell obtained from Le Bail fitting of the powder XRD data recorded at ambient temperature, with a difference in unit cell volume of 1.4%.

**Table 3:** Comparison of unit cell parameters for form III of *m*-ABA

	Unit cell parameters						Volume per molecule (Å^3^)
	*a*/Å	*b*/Å	*c*/Å	*α*/°	*β*/°	*γ*/°	
Le Bail fitting of powder XRD data recorded at ambient temperature^*a*^	3.777	7.296	21.339	90	94.8	90	146.50
Structure from precise geometry optimization^*b*^	3.733	7.325	21.177	90	93.8	90	144.43
Le Bail fitting of powder XRD data recorded at 70 K (Fig. 2)^*c*^	3.737	7.314	21.302	90	95.5	90	144.88

^*a*^Unit cell parameters from Le Bail fitting^12^ of the experimental powder XRD data recorded at ambient temperature.

^*b*^Unit cell parameters for the structure obtained following precise geometry optimization of the reported crystal structure of form III.

^*c*^Unit cell parameters from Le Bail fitting of experimental powder XRD data recorded at 70 K, starting with the unit cell of the structure obtained following precise geometry optimization of the reported crystal structure of form III.

Similarly, for form IV of *m*-ABA, successful Le Bail fitting of powder XRD data recorded at 70 K was achieved using, as input to the calculation, the unit cell of the reported structure of form IV following precise geometry optimization (Fig. S1, ESI†). However, successful Le Bail fitting of powder XRD data recorded at ambient temperature was not achieved using the same input unit cell.

**Fig. 3:**
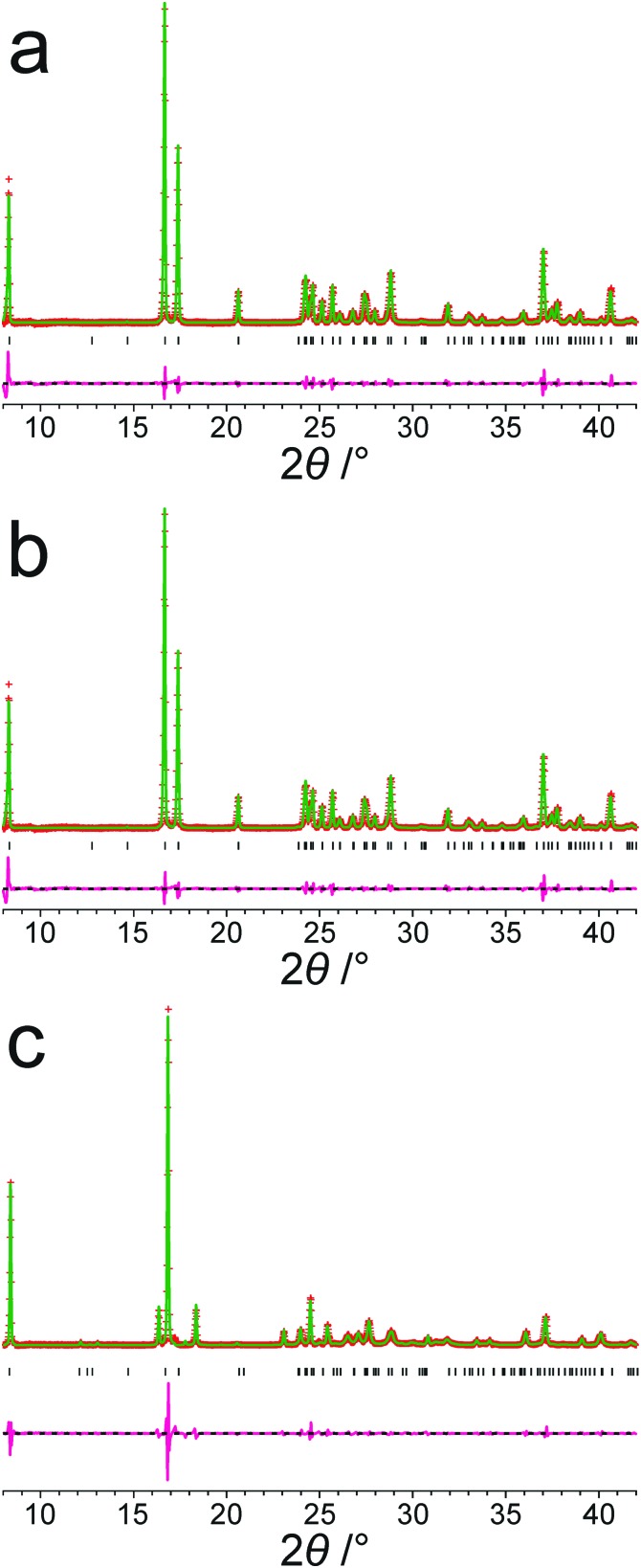
Results from Le Bail fitting of experimental powder XRD data recorded at 70 K: (a) initial unit cell from structure **1** and experimental data for form III, (b) initial unit cell from structure **2** and experimental data for form III, and (c) initial unit cell from structure **7_R_** and experimental data for form IV. In each case, the initial unit cell was taken from the structure after precise geometry optimization. The fitted unit cell parameters in each case are given in Table S2, ESI†. Red + marks, experimental data; green line, calculated data; magenta line, difference plot; black tick marks, predicted peak positions.

**Fig. 4:**
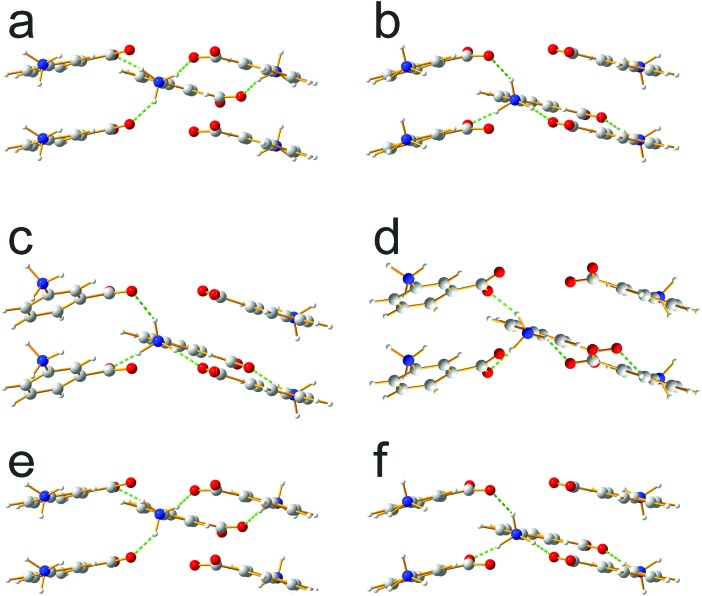
Comparison of intermolecular hydrogen bonding in (a and b) the reported crystal structure of form IV, (c and d) structure **7** from AIRSS and (e and f) structure **7_R_** from AIRSS, in all cases following precise geometry optimization. For each structure, the two views correspond to the two independent molecules in the asymmetric unit.

Focusing on the 11 structures of lowest energy from the AIRSS calculations (Table 2), Le Bail fitting to the low temperature (70 K) experimental powder XRD data for form III was successful for structures **1** and **2** (Fig. 3a and b). However, while the unit cells of structures **3**, **7** and **10** are similar to that of form IV (see Fig. 1b), Le Bail fitting against low-temperature powder XRD data was not successful for these structures. To investigate this issue further, careful inspection of structure **7** shows that, while each N–H bond of the NH_3_^+^ group is engaged in intermolecular N–H···O hydrogen bonding, the intermolecular hydrogen bonding is different from the reported crystal structure of form IV (Fig. 4), corresponding to rotation of the C–NH_3_^+^ group around the C–N bond. In this respect, structure **7** can be considered as a local metastable variant of form IV, and we expect that a longer AIRSS calculation would generate a structure with the same low-energy hydrogen-bonding network observed in the reported crystal structure of form IV. An alternative approach to find this structure would be to carry out a quenched molecular dynamics calculation, allowing structures to hop over any small local minima. However, the most computationally efficient way to generate the known structure of form IV is simply to rotate the C–NH_3_^+^ group in structure **7** by *ca.* 60° around the C–N bond, followed by DFT-D geometry optimization (which may be included automatically in the AIRSS procedure). Using this approach, a structure of lower energy (by 0.87 kJ mol^–1^) and higher density (the unit cell volume decreases from 148.26 Å^3^ to 146.10 Å^3^) was obtained and is denoted structure **7_R_**. It is clear from Fig. 4 (compare Fig. 4a and b with Fig. 4e and f) that the intermolecular hydrogen-bonding arrangement in structure **7_R_** is identical to that in the reported crystal structure of form IV. Using the unit cell of this modified structure as input, successful Le Bail fitting to the experimental low-temperature (70 K) powder XRD data of form IV was achieved (Fig. 3c). Simulated powder XRD patterns for structures **1** to **11** and **7_R_** from the AIRSS calculations and for the reported crystal structures of forms III and IV, following precise geometry optimization in all cases, are shown in Fig. S2 (ESI†).

In addition to powder XRD, solid-state NMR provides an alternative approach for assessing whether structures obtained from structure prediction calculations match a specific experimentally known polymorph. Within an NMR Crystallography approach, chemical shieldings for a given structure can be calculated for predicted structures using the GIPAW method,^45^,^47^,^50^–^52^ and then compared to experimental solid-state NMR data. Table 4 compares the calculated absolute isotropic shieldings (for ^1^H, ^13^C, ^15^N and ^17^O) for the structures obtained in the AIRSS calculations and the corresponding data calculated for the reported crystal structures of form III and form IV (in all cases following precise geometry optimization). In the case of ^1^H and ^13^C shieldings, the root mean squared deviation over all sites is specified (see Table S4, ESI† for the full list of calculated absolute isotropic shieldings). It is striking that the three cases for which successful Le Bail fitting was achieved using low-temperature powder XRD data [structure **1** (form III), structure **2** (form III) and structure **7_R_** (form IV)] are also the cases with the lowest differences in absolute isotropic shieldings between computed and experimental solid-state NMR data. The highest discrepancies in these cases are 0.08 ppm (^1^H), 0.10 ppm (^13^C), 0.13 ppm (^15^N) and 0.66 ppm (^17^O), whereas the lowest discrepancies for all other structures are 0.26 ppm (^1^H), 0.52 ppm (^13^C), 0.38 ppm (^15^N) and 1.94 ppm (^17^O). The significant difference in calculated NMR parameters between structures **7** and **7_R_** is particularly noteworthy. In spite of the close similarity between structure **7** and the reported structure of form IV (*e.g.*, see the COMPACK similarity measure^65^ in Table S1, ESI†), the changes in hydrogen bonding arising from rotation of the C–NH_3_^+^ group to convert structure **7** to structure **7_R_** are such that the computed NMR parameters of structure **7** are in poor agreement with the those for form IV whereas the computed NMR parameters of structure **7_R_** are in very close agreement with those for form IV. These observations illustrate the potential for the combined use of NMR crystallography and powder XRD data analysis to validate the link between structures generated in structure prediction calculations and the crystal structure of the material used to record the experimental data. The challenge, however, is that DFT-based methods for calculating solid-state NMR chemical shifts, such as GIPAW and other related methods for electronic structure calculations,^67^,^68^ do not give perfect agreement to experimental NMR data.

**Table 4:** Differences between the GIPAW calculated^*a*^ absolute isotropic shielding for the 11 lowest-energy crystal structures of *m*-ABA generated by AIRSS and the corresponding data calculated for the reported^12^ crystal structures of forms III and IV of *m*-ABA

Structures compared	Difference in absolute isotropic shielding/ppm			
	^1^H^*b*^	^13^C^*b*^	^15^N	^17^O
**1**/form III	0.05	0.07	0.02	0.05
**1**/form IV^*c*^	0.36	1.10	0.72	5.39
**2**/form III	0.08	0.10	0.06	0.29
**2**/form IV^*c*^	0.37	1.10	0.73	5.41
**3**/form III	0.36	1.47	2.64	3.78
**3**/form IV^*c*^	0.37	1.11	0.78	5.60
**4**/form III	0.33	0.52	0.39	1.94
**4**/form IV^*c*^	0.43	0.96	1.06	5.30
**5**/form III	0.34	0.53	0.38	2.01
**5**/form IV^*c*^	0.44	0.98	1.05	5.38
**6**/form III	0.31	0.56	0.44	2.05
**6**/form IV^*c*^	0.42	0.96	1.10	5.15
**7**/form III	0.27	2.33	1.19	23.49
**7**/form IV^*c*^	0.39	2.03	1.71	18.83
**7_R_**/form III	0.34	1.07	0.76	4.75
**7_R_**/form IV^*c*^	0.04	0.07	0.13	0.66
**8**/form III	0.87	1.80	2.23	14.14
**8**/form IV^*c*^	0.92	1.50	1.77	18.83
**9**/form III	0.96	1.78	1.02	13.56
**9**/form IV^*c*^	1.01	1.45	0.64	18.31
**10**/form III	0.26	1.01	1.41	4.11
**10**/form IV^*c*^	0.43	1.27	1.96	7.19
**11**/form III	0.37	1.57	1.81	7.91
**11**/form IV^*c*^	0.56	1.50	1.98	12.64

^*a*^After precise geometry optimization.

^*b*^Root-mean-squared deviation for all atoms in the *m*-ABA molecule.

^*c*^Root-mean-squared deviation for the two *m*-ABA molecules in the asymmetric unit.

**Fig. 5:**
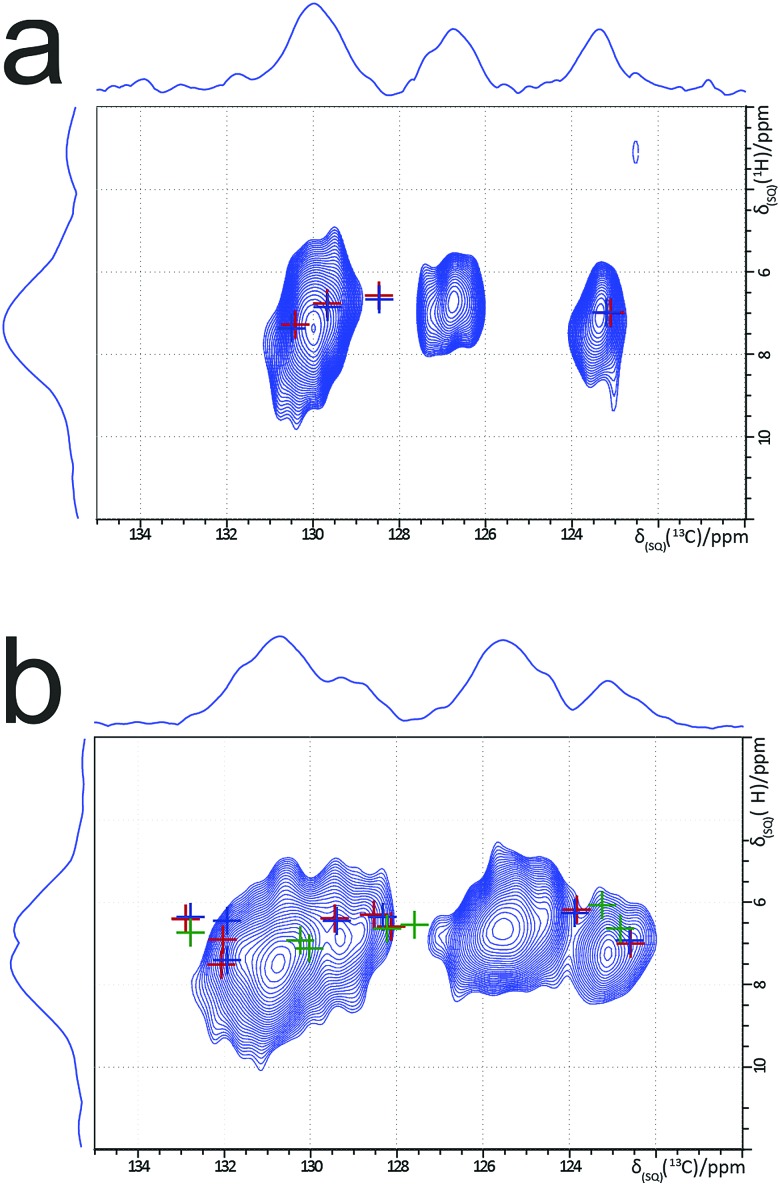
^1^H–^13^C (600 MHz) heteronuclear correlation spectra recorded for (a) form III and (b) form IV of *m*-ABA (MAS frequency, 60 kHz; CP contact time, 100 μs). Experimental conditions for (a): 50*t*_1_ FIDs with *t*_1_ increment 83.3 μs (using the States-TPPI method to achieve sign discrimination); 128 transients co-added for recycle delay 1.5 s; experimental time 2.7 h. The same experimental conditions were used for (b), except: 32*t*_1_ FIDs; 512 transients; experimental time 6.8 h. In (a), GIPAW calculated chemical shifts are indicated for structure **1** (blue crosses) and the reported crystal structure of form III (red crosses). In (b), GIPAW calculated chemical shifts are indicated for structure **7_R_** (blue crosses), structure **7** (green crosses) and the reported crystal structure of form IV (red crosses).

Experimental one-dimensional ^1^H–^13^C cross polarization (CP) MAS solid-state NMR spectra of forms I to V of *m*-ABA have been reported.^49^Fig. 5 presents experimental two-dimensional ^1^H–^13^C MAS solid-state NMR heteronuclear correlation spectra for form III and form IV of *m*-ABA. These spectra were recorded using a short cross polarization duration of 100 μs such that only the peaks corresponding to directly bonded C–H moieties are expected to be observed. In Fig. 5b, crosses show the GIPAW calculated ^13^C and ^1^H chemical shifts for the reported crystal structure of form IV (red), structure **7_R_** (blue) and structure **7** (green), in all cases following precise geometry optimization. In this way, Fig. 5b shows the differences between calculated ^13^C and ^1^H chemical shifts for structures **7** and **7_R_**, as discussed above in the context of Table 4. However, as observed in previous examples of two-dimensional ^1^H–^13^C correlation NMR spectra,^69^–^75^ only reasonable (not perfect) agreement is observed between experimental and GIPAW calculated ^13^C and ^1^H chemical shifts at the level of *ca.* 1% of the chemical shift range. As a consequence, we cannot reliably conclude whether structure **7** or structure **7_R_** gives the best agreement to the experimental NMR data. In line with previous observations,^76^,^77^ *m*-ABA is a challenging case in which there is only a small spread of experimental ^1^H chemical shifts.

It is well established that crystal structure prediction calculations typically generate many more energetically accessible structures than experimentally identified polymorphs.^28^,^78^ Within the 11 structures of lowest energy found from the AIRSS search in this study, at least three new structure types are identified that do not match any of the polymorphs of *m*-ABA that have been reported in experimental studies.^12^ We refer to these new structure types as **A** (corresponding to structure **3**), **B** (corresponding to structures **4**, **5** and **6**) and **C** (corresponding to structures **8** and **9**). All three of these structure types contain bilayers of *m*-ABA molecules, in common with the known forms III and IV. Following precise geometry optimization, the energy of structure type **A** is comparable to that of form IV and careful analysis of the structure reveals that it closely resembles form IV, but with different pairs of molecules related by inversion symmetry. Structure types **B** and **C** have somewhat higher energies and are structurally more distinct from the known polymorphs.

### Summary and outlook

This paper has shown that, within the 11 lowest-energy structures generated in a DFT-D based AIRSS search (restricted to centrosymmetric structures containing zwitterionic *m*-ABA molecules with *Z* = 4), predicted structures corresponding to the experimentally determined structures of form III and form IV of *m*-ABA are successfully obtained. It is noteworthy that these known crystal structures were found successfully among only 600 structures generated in the AIRSS search (we note that form IV, corresponding to structure **7_R_**, was found after an appropriate rotation of the NH_3_^+^ group in structure **7** from AIRSS, followed by further geometry optimization); this number is several of orders of magnitude lower than the typical number of trial structures generated in the commonly used methods for crystal structure prediction of organic materials.^19^,^22^–^31^

An important aspect of this work has been to assess the subsequent validation of the structures generated from AIRSS by comparison to experimental data, specifically the complementary methods of powder XRD and solid-state NMR. In the case of powder XRD data, it is found that the unit cell parameters of predicted structures that are identified as matching form III or form IV give rise to successful Le Bail fitting only for experimental powder XRD data recorded at low temperature (70 K), and not for experimental powder XRD data recorded at ambient temperature. Assessment of predicted structures against experimental NMR chemical shifts provides complementary insights, as the chemical shifts depend on the local environments of the atomic nuclei, and are therefore sensitive to specific structural features such as molecular conformation and intermolecular interactions (*e.g.*, hydrogen bonding, ring currents, C–H···π interactions and π···π interactions).^79^ In the above discussion, GIPAW calculated ^1^H and ^13^C chemical shifts for directly bonded C–H nuclei were compared to high-resolution experimental ^1^H–^13^C MAS NMR heteronuclear correlation spectra. While DFT-D predicted NMR chemical shifts do not enable direct identification of the crystal structure, it is evident from our discussion of structures **7** and **7_R_** that clear differences in calculated NMR chemical shifts arise from subtle changes in hydrogen bonding networks, thus providing complementary information to the assignment of structures based on comparison to powder XRD data. For more complex molecules, the NMR crystallography approach could be enhanced by using dynamic nuclear polarization (DNP) to allow both ^13^C–^13^C and ^13^C–^15^N two-dimensional correlation spectra to be recorded at natural isotopic abundance to enable assignment,^80^,^81^ and to observe ^13^C double-quantum build-up curves to probe intermolecular packing.^82^

This proof-of-principle study points to the feasibility of using AIRSS to generate structural models of energetically accessible polymorphs of organic materials, which may then be used as initial structural models for structure determination using a combination of powder XRD methods and NMR crystallography techniques (*e.g.*, in cases for which structure determination from powder XRD data alone is challenging). Recent work by Dudek *et al.*^83^ is also relevant in this regard. We note that none of the structures generated in our AIRSS calculations gave rise to a powder XRD pattern resembling the experimental powder XRD pattern of form I of *m*-ABA, which has so far eluded structure determination. Although our AIRSS search was carried out using the *m*-ABA molecule in the zwitterionic form (which is known from experimental studies^12^ to be the tautomeric form of the *m*-ABA molecule in form I), it is possible that other constraints imposed in our AIRSS search (specifically, centrosymmetric structures with *Z* = 4) may not be compatible with the crystal structure of form I.

Finally, it is relevant to note that crystalline amino acids in the zwitterionic form often exhibit dynamics of the ammonium (NH_3_^+^) group *via* a 3-site 120° jump motion about the C–N bond (in such structures, the ammonium group is usually engaged in three intermolecular N–H···O hydrogen bonds). Importantly, the occurrence of this motion is not revealed as disorder in the time-averaged crystal structure determined from diffraction data because the local symmetry of the dynamic process matches the local symmetry of the dynamic moiety. Solid-state NMR techniques have been exploited^84^–^88^ to establish the dynamic properties of the ammonium group reorientation in a wide range of crystalline amino acids, revealing that the rate of the 3-site 120° jump motion discussed above can differ markedly depending on the geometric details of the hydrogen-bonding arrangement involving the ammonium group. In the context of crystal structure prediction strategies of the type discussed in this paper, it is relevant to ponder the extent to which differences in the rotational frequency of the ammonium group may contribute to differences in the entropies of different crystal forms, which may influence the relative ranking (based on Gibbs free energies) of different crystal structures generated in the search.

## Conflicts of interest

There are no conflicts to declare.

## Supplementary Material

Supplementary information
