# Supplementary material for: Ab initio random structure searching of organic molecular solids: assessment and validation against experimental data
Source: Phys Chem Chem Phys. 2017 Sep 25;19(38):25949–60. doi: 10.1039/c7cp04186a (PMC5779078; doi:10.1039/c7cp04186a)
Supplement: Supplementary file 1 [file CP-019-C7CP04186A-s001.pdf]

Electronic Supplementary Material (ESI) for Physical Chemistry Chemical Physics.
This journal is © the Owner Societies 2017

Ab-Initio Random Structure Searching of Organic Molecular Solids: Assessment and Validation Against Experimental Data

Miri Zilka,# Dmytro V. Dudenko,# Colan E. Hughes, P. Andrew Williams, Simone Sturniolo, W. Trent Franks, Chris J. Pickard, Jonathan R. Yates,* Kenneth D. M. Harris,* Steven P. Brown*

Supporting Information

Table S1. Comparisons between the 11 lowest-energy structures obtained in the AIRSS calculation and the reported structures of forms III and IV of *m*-ABA.

Structure	Energy difference[a] (eV/ molecule)	Space group[b]	Similarity[c]	RMS[d] (Å)
1	–	$P2_1/c$	15 (to form III)	0.015
2	0.007	$P\bar{1}$	15 (to form III)	0.172
3	0.037	$P\bar{1}$	15 (to form IV)	0.379
4	0.039	$P\bar{1}$	14 (to form IV)	0.466
5	0.043	$P\bar{1}$	14 (to form IV)	0.468
6	0.043	$P\bar{1}$	14 (to form IV)	0.449
7	0.050	$P\bar{1}$	15 (to form IV)	0.321
8	0.053	$P\bar{1}$	11 (to form IV)	0.859
9	0.059	$P\bar{1}$	11 (to form IV)	0.806
10	0.070	$P\bar{1}$	12 (to form IV)	0.430
11	0.071	$P\bar{1}$	12 (to form III)	0.267

[a] Relative to the energy of structure 1.
[b] Determined using spglib (http://atztogo.github.io/spglib/) with a tolerance setting of 0.1 Å
[c] Determined using the COMPACK crystal structure similarity procedure[1] implemented in the CCDC Mercury software (see also discussion in section 2.3 of Ref. [2]) : 15 out of the 15 is the highest similarity score.
[d] The average root-mean squared difference over all molecules determined using CCDC Mercury software.

Table S2. Comparison of unit cell parameters for forms III and IV and AIRSS generated structures of *m*-ABA

	Unit cell parameters[a]						Volume per molecule (Å^3)
	a / Å	*b* / Å	*c* / Å	α / °	β / °	γ / °	
Published crystal structures, determined from powder XRD data at ambient temperature							
Form III	3.777	7.296	21.339	90.00	94.80	90.00	146.50
Form IV	3.800	11.554	14.633	110.50	92.70	96.60	148.78
Structures after *initial geometry optimization*							
Form III	3.711	7.310	21.048	90.00	93.42	90.00	142.52
Form IV	3.724	11.612	14.485	110.60	95.67	96.27	144.13
**1**	3.709	7.306	21.089	90.10	93.50	90.17	142.61
**2**	3.704	7.315	21.112	88.38	85.49	89.38	142.49
**3**	3.764	10.912	14.793	103.55	92.45	97.31	146.04
**4**	7.387	7.404	10.878	80.64	80.66	88.47	144.81
**5**	7.392	8.173	10.928	77.46	79.80	64.68	144.97
**6**	7.383	7.414	10.900	98.77	100.53	91.95	144.65
**7**	3.743	11.390	14.776	110.48	92.84	95.51	146.26
**8**	4.049	7.758	19.483	91.86	91.23	99.29	150.86
**9**	8.087	8.157	10.410	106.46	94.82	110.67	150.76
**10**	3.749	11.691	14.611	112.64	90.29	90.44	147.74
**11**	3.786	7.510	21.039	96.43	92.98	94.00	147.98
Structures after *precise geometry optimization* (see Table 2)							
Form III	3.733	7.325	21.177	90.00	93.82	90.00	144.43
Form IV	3.749	11.656	14.521	110.68	95.32	96.44	145.96
**1**	3.730	7.327	21.188	89.95	86.15	90.00	144.43
**2**	3.732	7.323	21.183	90.07	93.83	90.02	144.40
**3**	3.793	10.942	14.868	103.53	92.93	97.10	148.32
**4**	7.409	7.458	10.951	80.35	80.68	88.11	147.17
(*N* = 2)	3.729	7.409	10.951	80.68	80.35	88.11	147.17
**5**	7.455	8.183	10.958	77.55	79.98	64.77	146.99
(*N* = 2)	3.728	7.407	10.943	80.68	80.42	88.15	146.99
**6**	7.429	7.454	10.935	100.30	98.69	92.25	146.89
(*N* = 2)	3.727	7.429	10.904	80.51	80.65	87.75	146.89
**7**	3.752	11.513	14.712	109.88	90.85	96.32	148.26
**7_R**	3.744	11.716	14.484	110.61	95.45	96.66	146.10
**8**	4.104	7.814	19.525	91.58	91.56	100.02	154.01
(*N* = 2)	4.104	7.814	10.415	69.55	84.79	79.98	154.01
**9**	8.173	8.194	10.441	95.20	106.70	110.01	153.82
(*N* = 2)	4.097	7.789	10.441	69.57	84.80	80.39	153.82
**10**	3.761	11.774	14.740	66.87	88.65	88.05	149.95
**11**	3.816	7.549	21.125	96.46	93.20	94.52	150.37

Le Bail fitting to experimental powder XRD at 70 K (see Figures 2, 3 and S1)							
Form III	3.737	7.314	21.302	90.00	95.50	90.00	144.88
**1**	3.742	7.324	21.297	90.00	94.49	90.00	145.47
**2**	3.741	7.324	21.296	90.00	94.49	90.00	145.42
Form IV	3.744	11.624	14.480	110.47	94.78	96.25	145.48
$\mathbf{7_R}$	3.750	11.661	14.510	110.54	94.21	96.39	146.53

[a] Determined using tolerance setting of 0.1 Å, enabling a slight deviation from α = 90° and γ = 90° for structures with space group $P2_1/c$.

Table S3. Comparison of 2θ values (for $2\theta < 30°$) for the unit cell parameters of different structures representing form III of *m*-ABA.

Reported Structure [b]		*Geometry Optimized* [c]		*Le Bail Fit to powder XRD data recorded at 70 K* [d]	
2θ / °	{*h*, *k*, *l*}	2θ / °	{*h*, *k*, *l*}	2θ / °	{*h*, *k*, *l*}
8.309	{2,0,0}	8.362	{2,0,0}	8.334	{2,0,0}
12.817	{1,1,0}	12.780	{1,1,0}	12.793	{1,1,0}
14.713	{2,1,0}	14.703	{2,1,0}	14.702	{2,1,0}
16.663	{4,0,0}	16.769	{4,0,0}	16.713	{4,0,0}
17.429	{3,1,0}	17.453	{3,1,0}	17.435	{3,1,0}
20.656	{4,1,0}	20.714	{4,1,0}	20.679	{4,1,0}
23.618	{0,0,1}	23.965	{0,0,1}	23.905	{0,0,1}
24.204	{5,1,0}	24.281	{0,2,0}	24.242	{5,1,0}
24.38	{0,2,0}	24.295	{5,1,0}	24.319	{0,2,0}
24.394	{$\bar{2}$,0,1}	24.526	{$\bar{2}$,0,1}	24.562	{$\bar{2}$,0,1}
24.741	{1,2,0}	24.648	{1,2,0}	24.683	{1,2,0}

[b] Determined from powder XRD at ambient temperature

[c] After *precise geometry optimization* (see Table 2)

[d] After Le Bail fitting of the low-temperature (70 K) powder XRD data (see Figure 2) starting with the structure obtained following *precise geometry optimization*

Table S4. Calculated (GIPAW) absolute isotropic NMR shieldings (in ppm)

	NH_3	C2-H	C4-H	C5-H	C6-H	C1	C2	C3	C4	C5	C6	C7	N	O1	O2
**Form III**	20.1	23.1	23.6	22.9	23.4	34.6	46.9	39.1	41.5	39.6	40.3	-3.2	172.4	-12.6	-38.6
**Form IV**	20.0	22.9	23.6	22.4	23.5	34.7	47.6	38.9	42.0	38.7	37.9	-4.2	173.4	-16.6	-39.4
	20.1	23.8	23.4	23.6	23.0	35.6	46.5	39.4	42.4	41.2	38.7	-1.9	172.5	-20.0	-31.9
**1**	20.0	23.1	23.5	22.8	23.3	34.6	46.8	39.1	41.5	39.5	40.3	-3.3	172.4	-12.6	-38.6
**2**	20.0	23.1	23.5	22.8	23.3	34.6	46.8	39.1	41.4	39.5	40.3	-3.3	172.4	-12.5	-39.0
**3**	19.5	23.3	23.9	23.0	23.6	32.8	48.6	38.1	44.2	38.4	38.0	-2.2	175.8	-5.3	-38.4
	19.6	23.3	23.3	23.5	23.2	35.8	45.3	38.0	42.7	40.7	39.9	-3.0	170.8	-14.2	-39.8
**4**	20.1	23.4	23.4	23.4	23.7	35.5	47.0	39.6	41.7	39.9	39.7	-2.5	172.0	-12.1	-35.9
**5**	20.1	23.4	23.4	23.5	23.7	35.5	47.0	39.6	41.7	39.9	39.8	-2.5	172.0	-12.5	-38.8
**6**	20.1	23.4	23.3	23.4	23.7	35.5	46.9	39.7	41.7	39.9	39.6	-2.5	172.0	-11.0	-38.9
**7**	19.6	23.3	23.9	23.0	23.2	32.3	47.4	39.9	47.0	40.2	38.0	-6.8	171.3	-23.6	-47.3
	19.9	23.4	23.3	22.8	23.0	36.4	42.9	38.4	42.3	40.6	40.4	-2.9	173.7	-42.0	-4.8
**7_R**	20.0	23.0	23.6	22.5	23.6	34.6	47.6	38.9	42.2	38.8	38.0	-4.2	173.5	-16.4	-39.4
	20.1	23.7	23.3	23.5	23.1	35.7	46.4	39.4	42.3	41.2	38.8	-2.1	172.4	-19.1	-32.9
**8**	18.8	22.8	24.4	23.7	22.6	34.9	45.9	37.8	44.2	38.1	37.2	-2.3	174.6	4.6	-48.8
**9**	18.6	22.9	24.5	23.7	22.6	35.8	46.3	37.8	44.3	38.3	37.4	-2.4	173.4	3.4	-49.1
**10**	19.8	23.1	23.4	23.4	23.4	35.4	45.3	38.8	42.8	39.6	40.3	-3.1	171.0	-11.6	-39.0
	19.7	23.1	23.6	23.1	23.0	33.4	47.5	38.3	42.7	40.8	39.6	-1.3	173.8	-6.6	-33.1
**11**	20.2	23.4	23.3	23.1	23.2	35.4	46.6	39.3	41.9	40.1	39.2	-2.0	172.0	-14.3	-33.8
	19.1	22.9	23.7	23.2	23.4	35.2	46.0	37.7	46.2	39.3	38.6	-1.7	174.9	1.4	-43.8

Figures

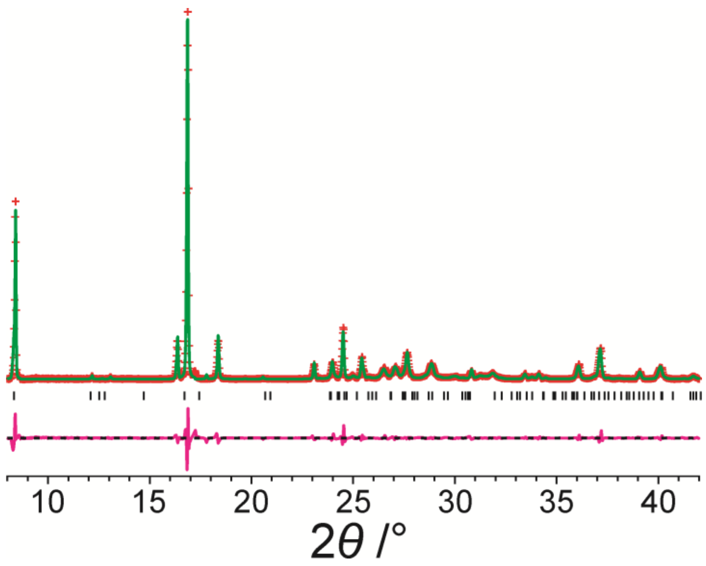

Figure S1. Le Bail fit (red + marks, experimental data; green line, calculated data; purple line, difference plot; black tick marks, predicted peak positions) of the experimental powder XRD pattern recorded at 70 K for form IV of *m*-ABA starting with the unit cell of the reported crystal structure following *precise geometry optimization*. The fitted unit cell parameters and unit cell volume are listed in Table S2.

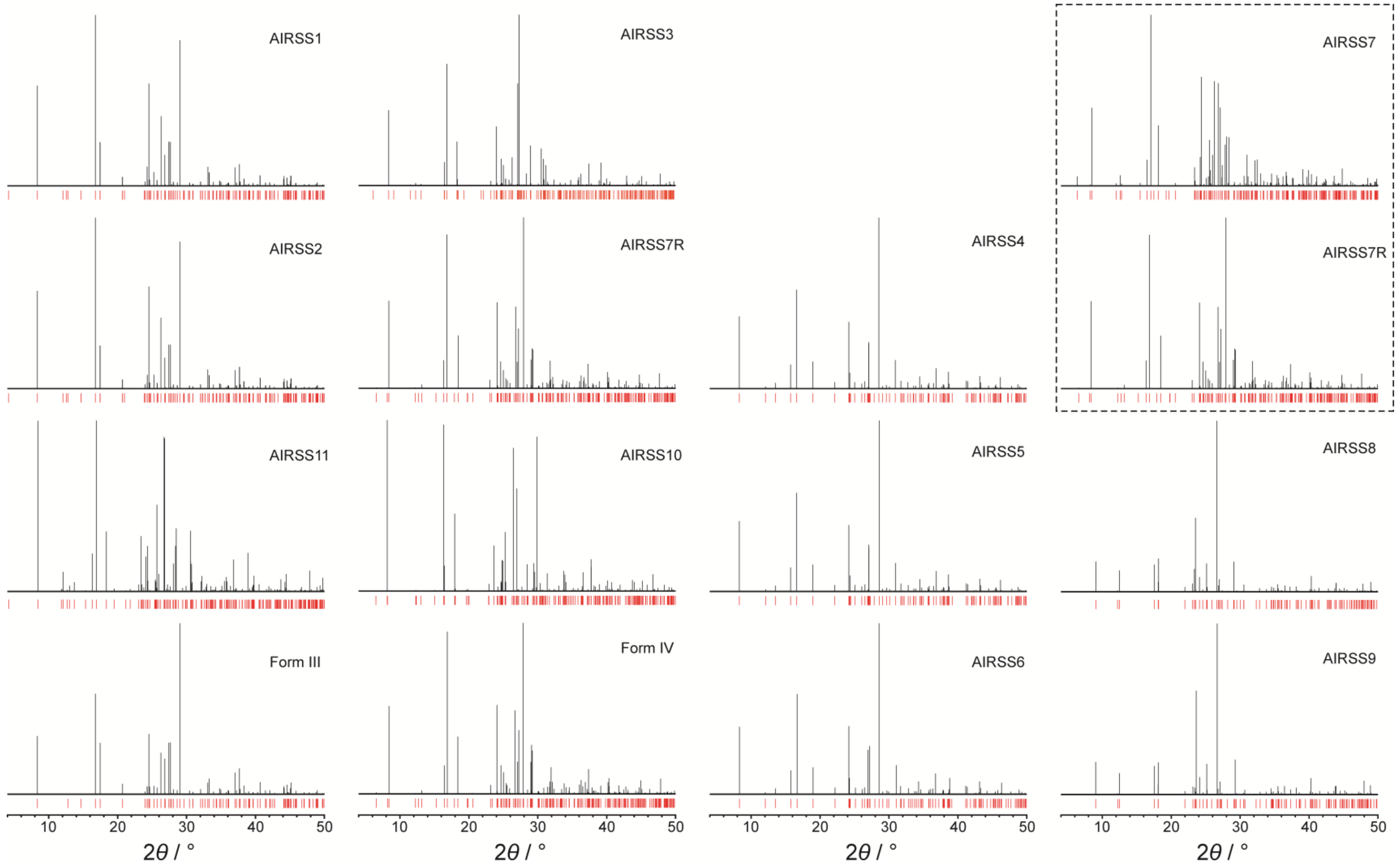

Figure S2. Simulated powder XRD patterns for structures **1**, **2**, **3**, **4**, **5**, **6**, **7**, **7$_R$**, **8**, **9**, **10** and **11** from the AIRSS calculations and for the reported crystal structures of form III and form IV, in all cases following *precise geometry optimization* (see Table 2). The organization into four columns corresponds to the groupings revealed in Figure 1b. The inset in the top right refers to comparison of structures **7** and **7$_R$**.

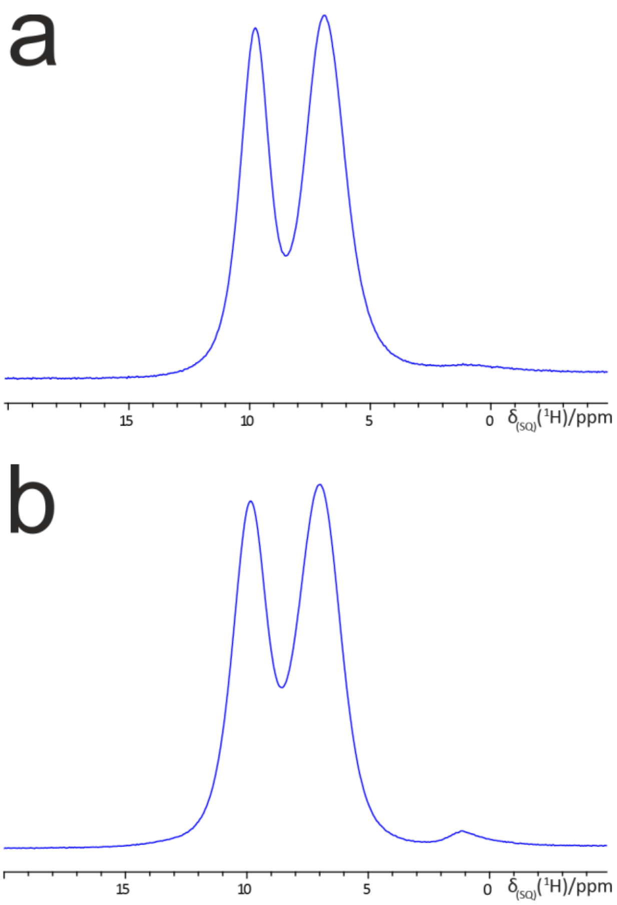

Figure S3. ^{1}H (600 MHz) one-pulse MAS (60 kHz) spectra of (a) form III and (b) form IV of *m*-ABA. In each case, two transients were co-added for a recycle delay of 2.5 s.

References

1. J. A. Chisholm and S. Motherwell, *J. Appl. Crystallogr.*, 2005, **38**, 228-231.

2. A. M. Reilly, R. I. Cooper, C. S. Adjiman, S. Bhattacharya, A. D. Boese, J. G. Brandenburg, P. J. Bygrave, R. Bylsma, J. E. Campbell, R. Car, D. H. Case, R. Chadha, J. C. Cole, K. Cosburn, H. M. Cuppen, F. Curtis, G. M. Day, R. A. DiStasio, A. Dzyabchenko, B. P. van Eijck, D. M. Elking, J. A. van den Ende, J. C. Facelli, M. B. Ferraro, L. Fusti-Molnar, C. A. Gatsiou, T. S. Gee, R. de Gelder, L. M. Ghiringhelli, H. Goto, S. Grimme, R. Guo, D. W. M. Hofmann, J. Hoja, R. K. Hylton, L. Iuzzolino, W. Jankiewicz, D. T. de Jong, J. Kendrick, N. J. J. de Klerk, H. Y. Ko, L. N. Kuleshova, X. Y. Li, S. Lohani, F. J. J. Leusen, A. M. Lund, J. Lv, Y. M. Ma, N. Marom, A. E. Masunov, P. McCabe, D. P. McMahon, H. Meekes, M. P. Metz, A. J. Misquitta, S. Mohamed, B. Monserrat, R. J. Needs, M. A. Neumann, J. Nyman, S. Obata, H. Oberhofer, A. R. Oganov, A. M. Orendt, G. I. Pagola, C. C. Pantelides, C. J. Pickard, R. Podeszwa, L. S. Price, S. L. Price, A. Pulido, M. G. Read, K. Reuter, E. Schneider, C. Schober, G. P. Shields, P. Singh, I. J. Sugden, K. Szalewicz, C. R. Taylor, A. Tkatchenko, M. E. Tuckerman, F. Vacarro, M. Vasileiadis, A. Vazquez-Mayagoitia, L. Vogt, Y. C. Wang, R. E. Watson, G. A. de Wijs, J. Yang, Q. Zhu and C. R. Groom, *Acta Crystallogr., Sect. B*, 2016, **72**, 439-459.
